# Adequacy of the Endometrial Samples Obtained by the Uterine Explora Device and Conventional Dilatation and Curettage: A Comparative Study

**DOI:** 10.1155/2014/578193

**Published:** 2014-01-08

**Authors:** Maria Abdulrahim Arafah, Ammar Cherkess Al-Rikabi, Rakia Aljasser, Yaser Adi

**Affiliations:** ^1^Department of Pathology, College of Medicine, King Saud University and King Khalid University Hospital, P.O. Box 7805, Riyadh 11472, Saudi Arabia; ^2^Department of Pathology, King Saud University, Faculty of Medicine and King Khalid University Hospital, P.O. Box 2925 (32), Riyadh 11461, Saudi Arabia; ^3^Department of Obstetrics and Gynecology, College of Medicine, King Saud University and King Khalid University Hospital, P.O. Box 7805, Riyadh 11472, Saudi Arabia; ^4^Sheikh Abdullah Bahamdan's Research Chair for EBHC-KT, King Saud University and King Khalid University Hospital, P.O. Box 7805, Riyadh 11472, Saudi Arabia

## Abstract

*Aims*. Our aim is to compare the adequacy and diagnostic yield of samples obtained by the endometrial Explora Sampler I-MX120 with endometrial specimens obtained by conventional dilatation and curettage (D&C). *Methods*. A total of 1270 endometrial samples were received in the histopathology laboratories at the King Khalid University Hospital, Riyadh, Saudi Arabia, between 2007 and 2010. In the outpatient clinic, the Uterine Explora Model I was used to obtain 996 samples. The remaining 274 samples were obtained by conventional D&C. Sample adequacy and the clustering of inadequate specimens according to age groups by the two different techniques were compared and statistically analyzed. *Results*. Out of 1270 endometrial samples, 253 (19.9%) were inadequate. The Uterine Explora was used in 88.5% of these inadequate samples (253 samples), and the remaining 11.5% were obtained by D&C. The insufficient tissue incidence was higher with the Explora (17.6%) than with the D&C (2.2%) and the difference was statistically significant (*P* < 0.0001). The ages of the patients, as well as the clinical indications for the procedures, were recorded. *Conclusion*. This retrospective study demonstrated better specimen adequacy when D&C was used compared to the higher rate of sample insufficiency obtained with the Explora.

## 1. Introduction

**Figure 1:**
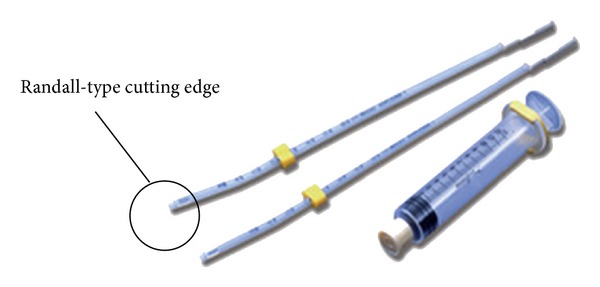
The Uterine Explora Model IMX120.

Abnormal uterine bleeding is one of the most common complaints presented to gynecologists. The majority of women with menorrhagia, postcoital bleeding, intermenstrual bleeding, or postmenopausal bleeding ultimately undergo diagnostic hysteroscopy with endometrial sampling as part of their assessment, particularly if symptoms persist or pelvic imaging suggests a uterine abnormality [1]. Dilatation and curettage (D&C) has been widely considered to be the method of choice for obtaining endometrial samples for histopathological evaluation. However, the needs for admission and general anesthesia and their associated costs have made this option less favorable [2]. In the outpatient setting, endometrial sampling is an effective and acceptable method for obtaining endometrial samples for histopathological assessment [3, 4]. However, approximately 10% of outpatient endometrial samples do not provide adequate tissue. Inadequate sampling is more problematic in postmenopausal women, for whom up to 68% of endometrial samples are reported to be inadequate [5]. In our institution, the only sampling tool available to perform the outpatient sampling procedure is the Uterine Explora Model I-MX120 (http://www.coopersurgical.com/) (Figure 1). This device utilizes a syringe technique in order to allow specimen recovery. In addition, the device is sterile and disposable (one-time use). The advantages of using Explora rather than D&C as a sampling device include a reduction in hospitalization costs, extra convenience for the patient and physician, and the minimal complications of the procedure. The purpose of this study is to compare the effectiveness of the Explora Model I tool with the conventional D&C technique for obtaining adequate endometrial samples that are capable of providing specific and informative histopathologic diagnoses.

## 2. Material and Methods

After obtaining the approval of our institutional review board, all endometrial samples received at the Histopathology Department in King Khalid University Hospital (KKUH, Riyadh, KSA) between January 2007 and December 2010 were included in this study. A total of 1270 endometrial samples were included (Table 1). Two hundred seventy-four samples (21.6%) were obtained by conventional D&C in the surgical theater, while the remaining 996 samples (78.4%) were obtained by senior obstetrics and gynecology residents who used a standardized biopsy technique in the outpatient procedure rooms. During the usage of the Explora Model I, the syringe provided with the instrument was used to create a negative pressure, and the Explora was rotated as it was withdrawn. After withdrawal, the tip was cut off, and the tissue was placed in 10% buffered formalin saline fixative and was sent for pathological examination. The endometrial samples were measured macroscopically and submitted in their entirety for processing. The pathologists who interpreted the endometrial samples were blinded to the instrument or method used to obtain the samples. All subsequent histopathology reports contained a comment on the adequacy of the specimen. An inadequate sample was defined as consisting of only blood, cervical mucus, endocervical epithelium, or blood with fragments of endometrial glands or stroma insufficient for histopathological assessment and diagnosis. The age, gravidity, parity, menstrual history, uterine size, hysteroscopy findings (when available), and the presence or absence of any cervical abnormality were recorded on the request forms, which were reviewed by the investigators. For each of the two methods used (Explora Model I and D&C), the numbers and percentages of inadequate samples and age group clustering were calculated and statistically analyzed. *P* values were determined when applicable.

## 3. Results

**Figure 2:**
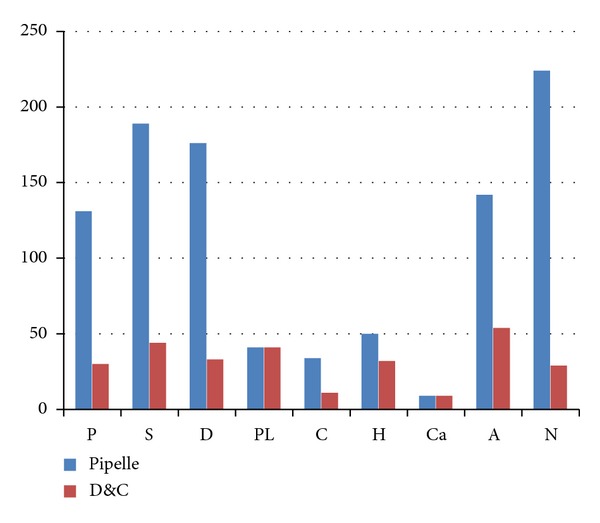
A diagram showing the distribution of different diagnostic categories between the two diagnostic methods (P: proliferative endometrium, S: secretory endometrium, D: disordered proliferative endometrium, PL: endometrial polyp, C: chronic endometritis, H: endometrial hyperplasia, Ca: endometrial carcinoma, A: adequate tissue with a combination of features e.g., an endometrial polyp and chronic endometritis, an endometrial polyp in a background of secretory or proliferative endometrium or atrophic endometrium, and N: nondiagnostic/insufficient).

**Figure 3:**
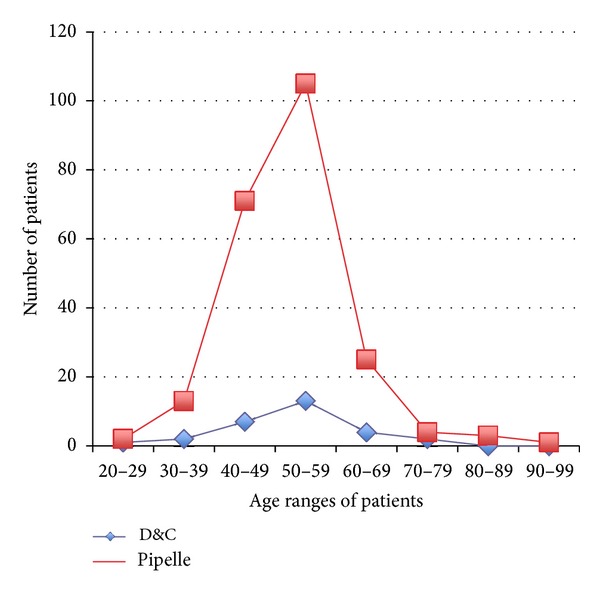
A diagram showing the age clustering of women with inadequate endometrial samples.

Of the 1270 endometrial samples obtained, 253 samples (19.9%) were scored as inadequate. Of these samples, the Explora sampler was used to collect 224 samples (88.5%), whereas 29 samples (11.5%) were obtained by D&C (Figure 2). Thus, the insufficient tissue percentage was higher with the Explora (17.6%) than with D&C (2.2%), which was a statistically significant difference (*P* < 0.0001). Age group clustering (i.e., numbers of premenopausal and postmenopausal women) of inadequate sample results was also calculated (Figure 3). Of the 253 inadequate samples, 82.6% were from women 45 years of age and older (i.e., postmenopausal) compared to 17.4% in premenopausal women; the age difference was significant (*P* < 0.0001). This finding was in agreement with those from other similar studies [5–8]. The detection rates of endometrial hyperplasia and carcinoma using both methods were assessed and calculated. Of the 73 samples with a diagnosis of endometrial hyperplasia, 50 (68.5%) were diagnosed by D&C, and 23 (31.5%) were diagnosed using the Explora sampler. This finding indicates a higher rate of detection for conventional D&C. However, of the 18 samples with a diagnosis of endometrial cancer, the rates of detection were similar between the two methods.

## 4. Discussion

Endometrial sampling for the evaluation of dysfunctional uterine bleeding and the diagnosis of endometrial hyperplasia and carcinoma and other indications remains one of the most commonly performed gynecological procedures [1–4]. In recent years, less hazardous and more inexpensive and convenient outpatient sampling methods have replaced the traditional, in-hospital, endometrial curettage. The advantages of outpatient endometrial biopsy include reduced cost and less risk for the patient, as no anesthesia is required. Furthermore, the discomfort and pain produced by sampling have been reported to be minimal [5]. However, it is essential to ensure that outpatient endometrial sampling is quantitatively adequate and comparably accurate to conventional dilatation and curettage. A sample is judged as adequate if a specific diagnosis can be given from the histological examination of the endometrial fragments obtained. Adequacy can be measured by comparison of either outpatient biopsy with curettage histological evaluation or outpatient biopsy with the results of pathological examination of hysterectomy specimens [3, 4]. Many techniques for obtaining an endometrial sample without the need for curettage have been described in the literature. These techniques include the Vabra aspirator tissue trap (Milex Products Inc., Chicago, IL, USA) and the Novak biopsy curette with a 10 mL syringe functioning as an aspiratory device, which have been shown to be equally effective compared to D&C in detecting an endometrial pathology [6–9]. However, the Vabra aspirator and Novak biopsy curette, although widely available and relatively inexpensive, have several disadvantages, including the need for an electric vacuum pump to perform the aspiration in the former technique and the pain caused by both methods [6]. As a result of these drawbacks, smaller inexpensive and self-contained instruments have been developed and the prototype of this class of endometrial samplers is the Pipelle. The Pipelle has been shown to have a diagnostic accuracy comparable to that of Vabra aspiration and the Novak curettage while causing less pain [9–11]. All of these instruments (i.e., the Vabra aspirator, the Novak biopsy curette, and the Pipelle) have low rates of false-negative and insufficient tissue results for the detection of endometrial abnormalities, as determined by comparison to hysterectomy specimens [11–13]. Furthermore, in a study by Huang et al. [14] it was found that Pipelle biopsy had a sensitivity of 99.2% in pinpointing high grade cancer and a sensitivity of 93% in detecting low grade malignancies; the sensitivities defined for D&C were 100% and 97%, respectively. While “excellent agreement” was generally noted between preoperative histology and grade and the final pathology, pre-operative endometrial sampling more commonly provided underestimates of final grade (low grade versus high grade) than overestimates. The Explora is somewhat similar in its design to the Pipelle, but clinical studies on its effectiveness are scarce, with the effectiveness ranging between 14.6 and 15% according to various studies [6, 15]. Our own findings revealed that the rate of obtaining inadequate samples using the Explora was much higher (17.6%) than the rates reported in the literature [6]. However, most of these cases (82.6%) were obtained from postmenopausal women with atrophic endometrial status. This finding is in keeping with the rates reported by other investigators [5–8, 16].

## 5. Conclusions

This retrospective study suggests that traditional D&C produces better endometrial sample adequacy than the Explora technique. This finding indicates that clinicians performing endometrial sampling would benefit from more experience and training using the Explora technique. Additional studies comparing the adequacy of samples obtained with different endometrial sampling techniques and devices are warranted. Furthermore, we recommend using the D&C procedure when the Explora-obtained samples are inconclusive or when the use of the Explora sampler is accompanied by ultrasound findings that are suspicious of endometrial hyperplasia or carcinoma.

## Figures and Tables

**Table 1:** Characteristics of patients on whom both Explora and D&C methods were used.

	Explora Model I *N* (%)	D&C *N* (%)	Significance level (*P* value)
Number of women	996	274	
Mean age (years)	48.1 (SD 8.3)	47.4 (SD 9.5)	*P* = 0.28
Median age (years)	48	47.5	
*Clinical indication *			
Menorrhagia	515 (52%)	108 (39%)	*P* < 0.0001
Postmenopausal bleeding	177 (18%)	60 (22%)	*P* = 0.11
Abnormal uterine bleeding	96 (10%)	32 (12%)	*P* = 0.26
History of thickened endometrium on ultrasound studies	84 (8%)	16 (6%)	*P* = 0.17
Postcoital/Postpartum bleeding	9 (0.9%)	2 (0.7%)	*P* > 0.9
Clinical history of endometrial polyp	15 (0.15%)	19 (7%)	*P* < 0.0001
Other clinical diagnoses	100 (10%)	37 (14%)	*P* = 0.2
*Histopathological diagnosis *			
Inadequate	224 (22%)	29 (11%)	*P* < 0.0001
Proliferative endometrium	131 (13%)	30 (11%)	*P* = 0.3
Secretary endometrium	189 (19%)	44 (16%)	*P* = 0.3
Disordered proliferative endometrium	176 (18%)	33 (12%)	*P* = 0.02
Endometrial polyp	41 (4%)	41 (15%)	*P* < 0.0001
Chronic endometritis	34 (3%)	11 (4%)	*P* > 0.9
Endometrial hyperplasia	50 (5%)	23 (8%)	*P* = 0.03
Endometrial carcinoma	9 (0.9%)	9 (3%)	*P* = 0.004
Other histopathologic diagnoses	142 (14%)	54 (20%)	*P* = 0.02

D&C: dilatation and curettage; SD: standard deviation, *P* value ≤ 0.05 is considered statistically significant.
